# Emerging Infections in Asia

**DOI:** 10.3201/eid1507.090450

**Published:** 2009-07

**Authors:** Peter Horby

**Affiliations:** Oxford University Clinical Research Unit, Hanoi, Vietnam

**Keywords:** Asia, HIV/AIDS and other retroviruses, SARS, paramyxoviruses, book review

Emerging Infections in Asia comprises a selection of scientific and historical review chapters on a variety of infectious diseases and includes contributors from diverse locations ranging from Saudi Arabia to Australia. The book arose from the editors’ experience with the emerging infections described and from their professional associations with the other contributors. The book is divided into 3 disease-specific sections that focus on avian influenza, severe acute respiratory syndrome (SARS), and HIV/AIDS, respectively; a fourth section contains short reviews of other infections. Because of the relatively small size of the book for such a broad topic (250 pages), each section covers only a few specific topics for each selected disease.

The book contains some well-written reviews and valuable narrative histories that are important to document (e.g., the 2003 SARS outbreaks in Singapore and Taiwan). The chapters on SARS in animals and emerging paramyxoviruses are particularly interesting, and the book includes topics (e.g., *Escherichia coli* and *Staphylococcus aureus*) that are often overshadowed by more glamorous emerging infections. However, it is disappointing in that the first 2 chapters contradict one another on the occurrence of person-to-person transmission of influenza virus A (H5N1), and the chapter on SARS in China could have been improved with more rigorous editing. Most chapters appear to have been written around 2006, and although they provide good snapshots of the state of knowledge at that time, readers will need to look elsewhere for more recent developments.

Emerging Infections in Asia also misses an opportunity to pull together the diverse topics and experiences discussed to provide new insights into the emergence of infectious diseases and into responses to the constantly shifting challenge of emerging infections. Recognizing this drawback, the editors say in their preface, “We hope that our book can help readers make their own conclusions and ask more questions.” Approached in this context, the book provides an account of the diversity and challenges of emerging infectious diseases in Asia and some informative historical reviews that will be of interest to students exploring this fascinating topic.

